# On the Use of Arsenic in Destroying the Sensibility of a Carious Tooth, Preparatory to Filling

**Published:** 1845-03

**Authors:** Edward Taylor

**Affiliations:** Natchez


*On the Use of Arsenic in Destroying the Sensibility of a Carious Tooth, Preparatory to Filling.* By Edward Taylor, M.D., Natchez. It is only when there is considerable inflammation and great sensibility in the carious bony structure of the tooth, that arsenic, or any other agent than an excavator, can be advantageously employed, and when, too, from the greater activity of the circulation, most danger is to be apprehended from so virulent a poison. But in the employment of it for this purpose, though it be applied under the restrictions recommended by Dr. Ide, in volume 2, page 247, of the American Journal of Dental Science, I regard it as an unsafe remedy, when the destruction of the pulp is not desired, and have discarded its use altogether, except in such cases.

There must always be more or less absorbed and taken into the general circulation, and the effects produced by it are in proportion to the quantity employed and the vascularity of the tooth. The irritation which it first excites in the part induces a determination of blood to it, and if the vascularity be great, the tooth will frequently become injected with red blood, which I have never known to happen in the treatment of a diseased tooth where arsenic had not been used; and I have no doubt that this will account for the injection of the tooth in the case mentioned by Dr. Norton in the last number of the fourth volume of the Journal. It may also serve as an answer to his first three inquiries. When the structure of the tooth is more dense and less vascular, several months will sometimes elapse before any inflammation will be evinced, and when once developed I have seldom been able to subdue it so as to preserve the pulp, and have often had the extreme mortification to see teeth, the health and beauty of which I had thought preserved, become a source of pain and annoyance.

The history of a single case may suffice to show its evil effects. About two years since I applied a small portion to the front incisor, (of a lady about 30 years of age,) of yellow, dense structure, for three or four hours. I then removed the diseased bone and filled it with gold, and received her hearty thanks for doing it with so little pain. In a few weeks it became subject to considerable pain upon the introduction into the mouth of anything hot, which, however, was borne with for some time, but finally it became so painful and discolored that she called for advice. The tooth was *very* purple, with severe throbbing. I applied several leeches and directed Seidlitz powder, which soon gave relief, but the next day it was as bad as ever, when the same treatment was observed with the same result. The day following it was as bad as before. I then removed the plug, and drilled to the nerve cavity, when a free discharge of blood ensued, which gave immediate and permanent relief so long as the opening remained. In a week or ten days I cleansed out the nerve cavity to near the end of the fang and filled it with gold, but in a day or two was obliged to remove the filling, when another free discharge of blood ensued, and afforded entire relief. I then introduced a tube, and filled the artificial cavity, which answered well, so long as the tube remained open; but during a short absence, it became obstructed, and an abscess was formed and discharged itself along the neck of the tooth. Thus, to avoid a few minutes' pain, she had suffered for days, and is disfigured by an unsightly tooth, which she must soon lose. A remedy of such specific action, without its injurious effects, would certainly be a desideratum; and if any of our professional brethren are acquainted with such an agent, I, for one, would be thankful to have them communicate it.

*American Journal of Dental Science.*

